# Patients' profile admitted to the ICU after establishment of a regulatory policy system for ICU patient allocation at public hospitals in Rio de Janeiro, Brazil

**DOI:** 10.1186/cc9887

**Published:** 2011-03-11

**Authors:** R Goldwasser, S Oliveira, C David, M Oliveira, A Babo

**Affiliations:** 1 Secretaria Estadual de Saude, Rio de Janeiro, Brazil; 2 UFRJ, Rio de Janeiro, Brazil

## Introduction

The consolidation of intensive care fundamentals was accompanied by growth of ICUs and increased utilization of intensive care services. Unfortunately it was not followed by national health planning. The demographic changing profile with a higher number of elderly patients and a changing case mix with less trauma patient admissions, associated with the high prevalence of cardiovascular diseases and the early approach to septic patients, will have implications on intensive care organization. A regulatory policy system for public ICUs was started in Rio de Janeiro to ensure appropriate selection and allocation of patients who need intensive care. The aim of this study is to report the profile of patients admitted to the ICU since the beginning of this new policy.

## Methods

A retrospective, 1-year, analysis of data from the Regulation Center. A nonchecklist medical application form is transmitted by fax for ICU patient allocation. Requests originated both from the hospital emergency room (HER) and nonhospital emergency units of care (Unidade de Pronto Atendimento (UPA)). The age, gender and the main prevalent diseases were recorded. Acute cerebrovascular disease (CVD) was considered all forms of stroke, both ischemic and hemorrhagic injuries; acute coronary disease (ACD) was considered stable and unstable angina and acute myocardial infarction; sepsis for severe sepsis and septic shock; trauma for any severe trauma, multiple trauma, burns and brain trauma; pneumonia for any severe lung infection with or without respiratory failure; and cardiac failure for any severe heart failure and acute pulmonary edema due to cardiac disease.

## Results

There were 15,036 applications, 10,360 (68.9%) forms from HER and 4,676 (31.1%) forms from UPA. From 12,591 adult requests, 7,333 were men and 5,258 were women. Mean age was 61.54 years old, and 461 (4%) were >80 years old. Major diseases that motivated the requests for admission were ACD (1.871, 15%), CVD (1.753, 14%), pneumonia with or without organ failure (1.678, 13%), sepsis (1.423, 11%), cardiac failure (825, 7%), trauma (741, 6%) and others (4,300, 34%).

## Conclusions

There was a significant number of ICU requests, mainly from in-hospital demand. The discussion regarding the indication of ICU care and knowledge of the patient profile may improve quality of the health critical care policy.

